# The Influence of Orthodontic Treatment Need on Oral Health-Related Quality of Life among 12–18-Year-Old Adolescents in Riyadh

**DOI:** 10.3390/healthcare10112153

**Published:** 2022-10-28

**Authors:** Nancy Ajwa, Arwa AlHammad, Luluh AlAmmar, Munira AlMarjan, Talal AlShugair, Leen AlManie, Durgesh Bangalore

**Affiliations:** 1 Preventive Dentistry Department, Orthodontic Division, College of Dentistry, Riyadh Elm University, Riyadh 13244, Saudi Arabia; 2 General Dentist, Ministry of Health, Riyadh 12233, Saudi Arabia; 3 College of Dentistry, Riyadh Elm University, Riyadh 13244, Saudi Arabia; 4 Dental Health Department, College of Applied Medical Sciences, King Saud University, Riyadh 11433, Saudi Arabia

**Keywords:** adolescents, DHC, IOTN, malocclusion, OHRQoL, oral health

## Abstract

This study assessed the prevalence of orthodontic treatment needs and oral health-related quality of life (OHRQoL) in 12–18-year-old adolescents and explored the association between OHRQoL and treatment needs, gender, education level and family income. A total of 243 participants with no prior history of orthodontic treatment were included in this cross-sectional study utilizing a standardized self-explanatory questionnaire and clinical examination. The questionnaire consisted of two parts. The first part included the participants’ demographics (age, gender, education level, economic status) and the second part contained the Arabic version of the oral health impact profile (OHIP)-14, which consists of 14 questions to assess the impact of the severity of malocclusion on routine activities. The clinical examination involved the dental health component (DHC) index of orthodontic treatment needs (IOTN). The outcome of the study showed that 46% of participants had little or no treatment needs, followed by 23.5% with borderline needs, and 30.5% with severe needs. Female participants had higher mean OHIP-14 scores (10.94 ± 8.17) compared to their male counterparts (8.44 ± 7.15), and the difference in the mean scores was significant (*p* = 0.015). The educational level did not significantly influence the mean OHIP-14 scores (*p* = 0.723), whereas the family income correlated negatively with the mean OHIP-14 scores. Participants with less family income had higher mean OHIP-14 scores (11.53 ± 8.67) compared to participants with high (8.22 ± 6.71) and average family income (10.68 ± 8.30). The mean OHIP-14 scores between the family income groups were statistically significant (*p* = 0.036). The overall OHIP-14 score of the participants was 9.67 ± 7.75. The need for orthodontic treatment is crucial among adolescents. It is recommended to have regular orthodontic consultations for adolescents and prompt referral for treatment to improve the OHRQoL.

## 1. Introduction

Malocclusion is the second most common oral health concern associated with a person’s social, functional and psychological well-being and compromising dentofacial aesthetics [1,2,3]. Accordingly, the oral cavity is considered an autonomous landmark in the human body that significantly impacts the overall quality of life [4]. According to oral health-related quality of life (OHRQoL), good oral health is no longer defined as the absence of oral illnesses and dysfunction. On the contrary, a positive sense of dentofacial self-confidence and the absence of detrimental effects of oral problems on social life are both included in OHRQoL [5]. For all that, it has been hypothesized that there is a direct relationship between the OHRQoL and orthodontic treatment needs among children and adolescents. Furthermore, socioeconomic status and education levels significantly impact OHRQoL [6,7].

There is a difference in the perception of oral disease and the evaluation of oral health between the dentist and the patient [8,9]. Understanding the effects of malocclusion on OHRQoL will help the patient to accept the orthodontic treatment beyond the clinician’s parameters [10]. Recently, clinicians have shed light on the importance of patient perception to understand their needs and satisfaction with the treatment and determine the overall quality of health [11,12]. OHRQoL can be the ideal measurement for treatment outcomes and needs, since social and psychological factors are considered motivations for patients to seek orthodontic therapy [13]. When identifying treatment needs, the oral health impact profile (OHIP) is frequently used as a reliable measurement, rather than a subjective clinical opinion [14]. 

Malocclusion prevalence varies widely by region and among different age groups and genders; yet, young populations, or adolescents, are still affected in large numbers among other populations [15,16,17]. Furthermore, a previous study has reported that 50% of adolescents and children worldwide suffer from one or another type of malocclusion. The reported malocclusion prevalence in previous studies have ranged from 39% to 93% [3,18,19], with the highest and lowest prevalence in Africa and Asia, respectively [18].

Malocclusion is assessed using a variety of indices. Brook and Shaw have developed the index of orthodontic treatment need (IOTN) as a scoring system for malocclusions [20]. Because of its simplicity, reliability and convenience, the IOTN is widely regarded as a system for determining orthodontic treatment needs. The IOTN evaluates actual and perceptive orthodontic treatment needs using two main components [21]. The first is the dental health component (DHC) composed of a five-grade index. The second is the aesthetic component (AC) which comprises of a 10-grading system of colour photographs that illustrate different levels of dental attractiveness [22]. 

The assessment of orthodontic treatment needs using the IOTN-DHC in different countries has reported a prevalence rate of 21.3% to 71.6% [22,23,24,25,26]. However, due to improved oral health care facilities in different countries, the prevalence of orthodontic treatment needs may decline. In order to gain comprehensive data and knowledge regarding the prevalence of different types of malocclusions and the need for orthodontic treatment, epidemiological research on malocclusion is necessary on a regular basis. This data can be utilized to establish and implement public dental health policies for orthodontic treatment in each country, and manage the available resources in these regions [27,28,29,30]. It is assumed that the severity of the malocclusion is always related to treatment needs. This assumption could be well accepted when treatment need is estimated for larger population groups [29].

The present cross-sectional study was conducted to assess the prevalence of orthodontic treatment need and OHRQoL in 12–18-year-old adolescents and to explore the association between OHRQoL and treatment need, gender, education level and family income among adolescents who attended orthodontic screening clinics at the College of Dentistry, Riyadh ELM University, Saudi Arabia. The null hypothesis is that the OHRQoL is not significantly influenced by the orthodontic treatment needs and demographic characteristics (gender, education level and family income) of the study participants.

## 2. Materials and Methods

The present cross-sectional, multi-centre study was conducted in June–July 2021 using a standardized self-explanatory questionnaire and clinical examination.

### 2.1. Ethical Aspects and Study Participants

This study was conducted in accordance with the ethical principles outlined by the World Medical Association Declaration of Helsinki, 1964, as subsequently revised in 2013. The Institutional Review Board (IRB) of Riyadh Elm University (REU), Riyadh, Saudi Arabia, reviewed the study protocol and provided the ethical clearance (“SRP/2021/51/445/452”). Furthermore, the participants and their parents or guardians were informed about the study’s purpose and the clinical examination. They were ensured that confidentiality of all the information reported in the study would be maintained. Signed informed consent was obtained from the participants, or their parents or guardians, before the commencement of the study.

A total of 243 participants aged 12 to 18 years and residing in Riyadh city were enrolled in the present study following strict inclusion and exclusion criteria. The inclusion criteria were participants who were medically fit, free from chronic periodontal conditions (community periodontal index score of <3) and untreated dental caries and signed the consent form. Participants with chronic medical conditions and/or systemic conditions, undergoing and/or undergone orthodontic treatment, craniofacial anomalies such as cleft lip and palate, mental disability, TMJ disorders and arthritis were excluded from the study [31]. 

### 2.2. Questionnaire Formulation and Reliability

The questionnaires were handed over to the participants during their regular orthodontic screening. The data was collected through specifically designed free-access Google forms that assist in data encoding and patient communication. It comprised of two sections. The first section included questions about the participant’s demographic and general details, including age, gender, educational level and family income [27]. The general authority of statistics in Saudi Arabia has determined an average family income of 10,000 Saudi Arabian Riyal (SAR) per month [32]. Accordingly, the economic status was categorised into high, average and low income. 

**Figure 1:**
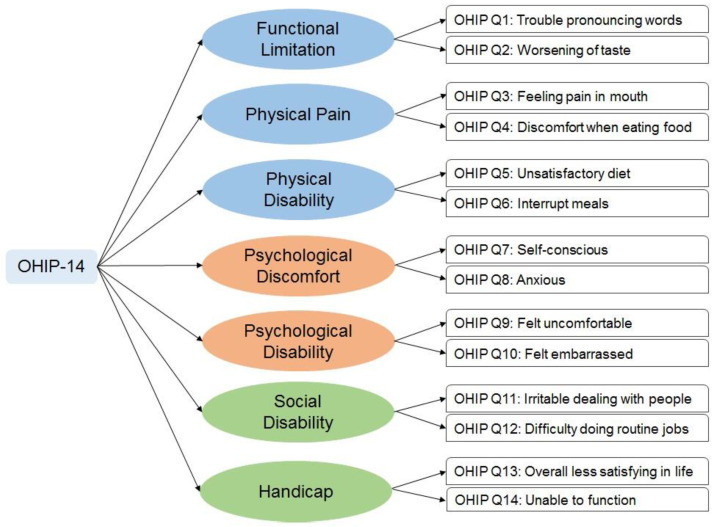
Oral Health Impact Profile-14 (OHIP-14) questions used in the study.

The second section contained questions related to the OHRQoL based on the oral health impact profile (OHIP)-14 questionnaire. The questions were framed to determine the impact of the severity of malocclusion on seven domains, each containing two items related to routine activities of the participants [33]. The domains were functional limitation, physical pain, physical disability, psychological discomfort, psychological disability, social disability and handicap (Figure 1) [21].

Each participant was asked about the frequency that he or she experienced an impact on 14 routine activities. The responses were recorded on a five-point Likert scale (0-never, 1-hardly ever, 2-occasionally, 3-fairly often and 4-very often) [31]. The responses to all 14 questions were summed up to determine the overall OHIP-14 score, which ranges from 0 to 56. The higher the OHIP-14 score, the worse the OHRQoL [34].

The presence of an impact was defined as the responses ‘occasionally’, ‘fairly often’ and ‘very often’, and for those without impact, the responses, ‘hardly ever’ and ‘never’ applied [35].

The questionnaire was initially framed in English and translated into Arabic by two independent professors fluent in English and Arabic at the Department of Preventive Dentistry/Orthodontic Division, Riyadh Elm University. The two translations were compared, and necessary editing was applied to the final draft. Subsequently, the final draft was translated back into the Arabic language by a translator. The translated versions obtained from the professors and translator were compared and evaluated by another professor, and a few adjustments were made to the final Arabic draft. The face validity of the final questionnaire was evaluated using Cohen’s Kappa Index, and a Kappa (κ) of >0.85 material validity ratio (CVR) was found, indicating good agreement. 

A pilot study was conducted with 25 patients aged 12–18 years to test the methodology and reliability of the questionnaires. The sample participants had no issues answering the questions, and the survey was completed in an average of 5–8 min. Reliability was assessed using inferential Cronbach alpha analysis, which demonstrated a value of 0.85, indicating good reliability. This outcome indicated that there was no need to change the proposed methodology. The patients in the pilot study were not included in the final study.

### 2.3. Clinical Examination and Examiner Reliability

The clinical examination was performed under artificial illumination in a dental chair using personal protective equipment, mouth mirror and CPI probes in accordance with WHO oral health survey guidelines [36,37]. Four examiners trained (theoretical and clinical) and calibrated for determination of the presence and severity of malocclusion using the IOTN-DHC performed the clinical examination [38]. 

The IOTN-DHC assessed 10 traits of malocclusion that included Class I, Class II and Class III buccal occlusions, overjet, reverse overjet, open bite, cross bite, overbite, impeded eruption, crowding, defects of cleft lip and palate as well as any craniofacial anomaly and hypodontia [20]. The treatment needs were categorized as little or no treatment need (grades 1 and 2), borderline need (grade 3) and treatment required (grades 4 and 5). The data recorded by the trained examiners were compared by an experienced orthodontist (N.A). Interrater (between the examiners A and B and an experienced orthodontist) and intrarater (two examinations by each independent with a one-week interval between examinations) agreement was determined using kappa (K) coefficients, with K = 0.92 and 0.94, respectively. 

### 2.4. Statistical Analysis

The Statistical Package for the Social Sciences (IBM^®^ SPSS^®^ Inc., Armonk, NY, USA) version 25 for Windows was used for processing and analyzing the data. Gender, educational level, family income and the IOTN were the independent variables, and OHRQoL was the dependent variable for the analyses.

Descriptive statistics were calculated for demographic variables, IOTN-DHC, and OHIP-14. Non-parametric Mann–Whitney U and Kruskal Wallis tests were applied to assess the significant differences in the mean overall OHIP-14 score among different IOTN groups and demographic variables, 

The impact of OHIP-14 on IOTN-DHC concerning demographic characteristics was analyzed and presented as a percentage of the occurrence. 

The chi-square analyses were performed to compare the participants’ demographic characteristics according to the IOTN and the impact of OHIP-14 in relation to demographic characteristics. For all the tests, the statistically significant difference between the measurements was determined at *p* < 0.05.

## 3. Results

A priori sample size calculation was applied to determine the minimum sample required for the study using the proportion estimate. Assuming that 50% of the subjects in the population have the factor of interest and a population size of 600, the study would require a sample size of 235 for estimating the expected proportion with 5% absolute precision and a 95% confidence level. A total of 248 participants met the inclusion criteria and were enrolled in the study. Of these, five participants did not attend the clinical examination and were excluded from the study, demonstrating a response rate of 97.98%. 

Table 1 presents the frequency and percentage distribution of the IOTN by demographic characteristics of the study participants. The mean age of the participants was 14.33 ± 2.18, with males and females representing 51% and 49% of the total participants, respectively. The chi-square test revealed no significant difference in the distribution of the IOTN-DHC by demographic characteristics of the study participants (*p* < 0.05).

**Figure 2:**
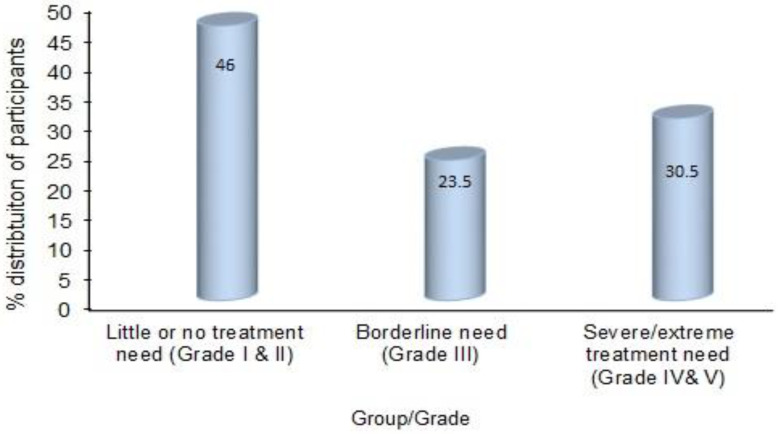
Distribution of the participants according to IOTN-DHC.

In this study, there were 112 participants with little or no treatment need (grades I and II of the IOTN-DHC), 57 with borderline need (grade III of the IOTN-DHC) and 74 with great or severe orthodontic treatment need (grades IV and V of the IOTN-DHC). The corresponding percentages were 46%, 23.5% and 30.5%, respectively (Figure 2).

Table 2 presents the frequency and percentage distribution of IOTN-DHC grades/subgrades among the study participants. The highest incidence of orthodontic severity was crossbite (21.4%), followed by openbite (20.2%), and the least severe was submerged deciduous teeth (0.4%).

The comparison of the mean OHIP-14 score across the demographic variables is presented in Table 3. Female participants had higher mean OHIP-14 scores than their male counterparts and the difference in the mean scores was significant (*p* = 0.015). The educational level did not significantly influence the mean OHIP-14 scores (*p* = 0.723). Family income correlated negatively with the mean OHIP-14 scores. Participants with less family income had higher mean OHIP-14 scores than participants with high and average family incomes. Moreover, participants with an average family income reported higher mean OHIP-14 scores than high family income participants. The mean OHIP-14 scores between the family income groups were statistically significant (*p* = 0.036).

Table 4 presents the mean score of the OHIP-14 items of the study participants. The mean score was high for OHIP-3 (1.53 ± 1.18; feeling pain in the mouth) in the physical pain domain, and the low score was for OHIP-Q12 (0.25 ± 0.66; difficulty doing routine jobs) in the social disability domain. The high and low mean OHIP-14 scores represented the greatest and least impact of the malocclusion on the study participants. 

Table 5 displays the comparison of mean OHIP-14 scores among different IOTN-DHC groups. When analyzing individual OHIP-14 items, the outcome revealed a significant difference only in the mean scores of OHIP Q7 (*p* = 0.019; feeling self-conscious) in the psychological discomfort domain, and OHIP Q9 (*p* = 0.023; felt uncomfortable) and OHIP Q10 (*p* = 0.002; felt embarrassed) in the psychological disability domain. The overall mean OHIP-14 score increased with treatment need (7.92–11.54), and the overall mean OHIP-14 score among different IOTN-DHC groups revealed a statistically significant value (*p* = 0.006). 

Among the participants, 81.5% of female orthodontic patients were significantly impacted by discomfort when eating food (OHIP Q4) compared to 33.3% of male counterparts in the borderline need group (*p* < 0.001). Similarly, a significant number of female participants (67.6%) were impacted by anxiety (OHIP Q8) compared to males (37.8%) in the treatment-required group (*p* = 0.010) (Table 6).

Table 7 presents the frequency of impact (in %) of each item of the OHIP-14 on the IOTN-DHC in relation to the educational level of the study participants. The college participants were least impacted by the OHIP-14, followed by high school and secondary school participants in all three IOTN-DHC groups. However, signiifcant differences were only observed for OHIP Q1, OHIP Q3, OHIP Q5, OHIP Q6 and OHIP Q11 between the different educational level groups in the borderline need category of IOTN-DHC (*p* < 0.001). 

Table 8 presents the frequency of impact (%) of each item of the OHIP-14 on IOTN-DHC in relation to the family income of the study participants. The participants with high family income were least impacted by the OHIP-14, followed by participants with average and low income in all three IOTN-DHC groups. However, significant differences were observed for OHIP Q2 (*p* < 0.008) in the little or no treatment required group, OHIP Q1 (*p* < 0.031) in the borderline need group, and OHIP Q13 of the treatment required IOTN-DHC group (*p* < 0.007).

## 4. Discussion

This cross-sectional study assessed the prevalence of orthodontic treatment needs and OHRQoL in 12–18-year-old adolescents among adolescents who attended orthodontic screening clinics at the College of Dentistry, Riyadh ELM University, Saudi Arabia. Furthermore, we also explored the influence of OHRQoL on the treatment needs, gender, education level and family income of the study participants. The study outcome demonstrated that OHRQoL was significantly influenced by orthodontic treatment need, gender and family income. In contrast, education level did not influence the OHRQoL of the study participants, thereby suggesting partial rejection of the null hypothesis.

The most common motive for seeking orthodontic treatment is to improve dental aesthetics and self-esteem. As a result, orthodontists should know that young patients may expect orthodontic treatment to improve dental health and function, appearance, self-esteem and social life [39]. Although, it is well-known that malocclusion has physical and psychological implications, research on the extent of these effects is still inconclusive. This could be owing to differing views of these impacts and a lack of consistent assessment methods [40]. Prevalence studies on the orthodontic treatment needs of children and young adults should be carried out regularly. This is significant since oral public health care is a major global priority for children and young adults. Additionally, in areas with limited resources, patient-based oral health outcomes can ensure that treatments are focused on the conditions most likely to have a detrimental influence on OHRQoL.

The IOTN–DHC outcomes in the present study demonstrated that 46% of patients represented the little or no treatment group, followed by 23.5% with borderline needs and 30.5% with definite orthodontic treatment needs. The overall mean OHIP-14 score significantly increased with treatment needs (7.92–11.54) thus demonstrating that the OHRQoL negatively impacted the participants requiring definite orthodontic treatment. Studies conducted in various parts of Saudi Arabia have provided conflicting results. In the studies of Alhummayani et al. [41] and Gudipaneni et al. [28], 24.3% and 21% of adolescents in the country’s western and northern regions had severe/extreme orthodontic treatment needs, respectively. On the contrary, Al-Jobair et al. [42], in their retrospective study, revealed that 70% of patients undergoing orthodontic treatment at King Saud University in the country’s central region had severe/extreme orthodontic needs. Similarly, Hassan et al., in 2006 [22] and 2014 [43], found that 71.6% and 52.5% of patients seeking orthodontic treatment at King Abdulaziz University hospital in the country’s western region were in severe or extreme need, respectively. The global IOTN-DHC prevalence rates have varied between 19–62.7% [23,41], with the lowest and highest prevalence rate in Yemen [44] and Iran [45], respectively.

The difference in the IOTN-DHC outcomes between the current and previous studies could be attributed to varying research design, demographic variables, sample selection (general population or orthodontic patients), study locations (hospitals, universities or schools), and referring dentists in addition to ethnic and cultural backgrounds [23,41].

True to this fact, Saudi Arabia observed a significant decline from 2006 to 2021 in the percentage of patients with severe/extreme orthodontic needs. This could be attributed to increased awareness of the impact of malocclusions on oral health and a better understanding of what orthodontic treatment can do to improve oral health and appearance. This has prompted parents to seek early interceptive and preventive orthodontic treatment [42].

The current results showed that the highest incidence of orthodontic problems were for crossbite (21.4%), followed by open bite (20.2%), and the least was submerged deciduous teeth (0.4%). A study done in the western region of Saudi Arabia mentioned similar results concerning open bite (20%), but the rate of crossbite was higher (44.5%) [22]. This may indicate that crossbite may be a characteristic of the Saudi population. The global prevalence rate of crossbite is reported to be between 5% and 22% [46]. The percentage of crossbite incidence in the present study is higher than those reported earlier; 9.5% by Bilgic et al. [47], 8% by Burden et al. [48], 12.2% by Ciuffolo et al. [49], 4.6% by Thilander et al. [50] and 14.2% by Perillo et al. [30].

Oral health related quality of life is a multi-dimensional concept that encompasses physical, social and psychological elements [5]. Clinical examinations do not analyze these aspects; instead, they primarily assess the presence and severity of the disease, rarely taking into account the impact of symptoms on quality of life [51]. The assessment of OHRQoL aids in the transition from traditional medical standards to assessment and care that places a greater emphasis on a person’s social and emotional experiences, as well as physical functioning; this establishes acceptable goals and, as a result, the outcome of therapy and treatment processes [52]. Questionnaires that collect data on oral health and the effects of oral health on quality of life are used to assess OHRQoL. In that context, the oral health impact profile (OHIP-14) questionnaire is used to assess the influence of oral health on OHRQoL [53]. Although OHIP-14 was developed with the intent of assessing OHRQoL in the elderly, it has now been accepted as a consistent and effective method for assessing OHRQoL in adolescents and young adults [53].

The overall OHIP-14 score recorded for the participants in this study was 9.67 ± 7.75. Thus, it can be inferred that the influence of malocclusion on participants’ daily activities was of low intensity as the mean value of OHIP-14 was <14, suggesting no impact on participants’ OHRQoL [54]. The low OHIP-14 score in the present study could be related to critical elements such as low frequency, low severity and a participant’s inability to recognize oral issues. Furthermore, the low score may be due to the low incidence and severity of adverse oral disorders that negatively affect OHRQoL in this age group, such as periodontal disease or tooth loss [55,56]. The mean score was high in the psychological disability domain, inferring that the participants with malocclusion had increased feelings of embarrassment and uncomfortableness in public places. This outcome is in agreement with previous studies reporting similar findings [33,45,57,58]. However, the low OHIP-14 scores in the social disability domain meant that participants had no problems doing routine jobs or dealing with people.

Because of their potential associations with the outcome and explanatory variables, this study recorded and compared demographic characteristics such as gender, educational level and family income [31]. Female participants had significantly higher mean OHIP-14 scores compared to male adolescents. When analyzing the OHRQoL impact, female adolescents demonstrated higher OHRQoL impact than males, especially for anxious feelings in the definite orthodontic treatment need group. This could be related to the higher anxiety of females about their physical appearance than males, who are less self-conscious [59,60,61,62]. According to de Oliveria and Sheiham [63], gender strongly influences how malocclusion affects OHRQoL, with women 1.22 times more likely than males to experience this problem. The present study agrees substantially with the previous studies demonstrating higher OHRQoL impact in females compared to males [31,64,65].

The education level of the participants in the current study did not significantly affect the OHRQoL. Participants with college education demonstrated non-significantly low OHIP-14 scores compared to other education groups, which could be attributed to increased self-awareness and self-esteem with higher education [31]. In addition, the end of the growth spurt during college lengthens the arch and provides more space for detention while reducing malocclusion concerns [66]. Furthermore, this could result from a “response shift” as a person ages; the longer an individual experience malocclusion, the more likely it is that they will get used to the limitations it imposes on their activities, which will minimize its impact [31]. The present findings disagree with the outcome of the previous study by Masood et al. [31], where participants with a university education had significantly higher impact on OHRQoL compared to participants with only secondary education [31].

The family income of the participants in the current study significantly influenced the OHRQoL. Patients with low family incomes showed higher scores of OHIP-14. This is in accordance with a study conducted in Sweden that demonstrated low income patients had inferior OHRQoL [67]. Similarly, Guimarães et al. [68] reported a significant difference in OHRQoL between economically underprivileged and economically more privileged participants. Children born into low socioeconomic level families lack access to early screening and preventive measures because of their low income.

The current study is bound by some methodological limitations that should be considered before generalizing the outcomes. As the present study’s main aim was to determine how various orthodontic treatment needs influenced the quality of life for young adult orthodontic patients, the participants were all patients who felt the need for treatment. They did not represent the overall young adult population, which may have diverse effects on routine activities according to the severity of malocclusion and the necessity for orthodontic treatment. The findings of this study may not be relevant in older patients as the physical attractiveness factor is more prevalent in the younger population. Future studies should focus on nationwide surveys and include participants from the general population with different age groups to give a broader picture of OHRQoL.

## 5. Conclusions

Based on the limitations of the present study, the following conclusions are drawn:

- The influence of malocclusion on participants’ daily activities was of low intensity as the mean value of OHIP-14 was <14, suggesting no significant impact on participants’ OHRQoL.
- OHRQoL was significantly influenced by IOTN-DHC, gender and family income of the participants.
- The psychological disability domain demonstrated higher OHIP-14 scores than other domains, thus inferring that the participants with malocclusion had increased feeling of embarrassment and discomfort.
- Female participants had significantly higher mean OHIP-14 scores than male adolescents, thus justifying the gender differences influencing OHRQoL.
- Free orthodontic consultation and treatment are highly recommended for children and adolescents to enhance the OHRQoL and overcome the expected psychological status of individuals at later stages.

## Figures and Tables

**Table 1:** IOTN-DHC by demographic characteristics of the study participants (N = 243).

Variables	Categories	Little or No Treatment Need	Borderline Need	Treatment Required	*p*-Value *
		n (%)	n (%)	n (%)	
Gender	Female	55 (49.1)	27 (47.4)	37 (50)	0.956
	Male	57 (50.9)	30 (52.6)	37 (50)	
Educational level	Secondary school	66 (58.9)	37 (64.9)	40 (54.1)	0.731
	High school	34 (30.4)	16 (28.1)	27 (36.5)	
	College	12 (10.7)	4 (7)	7 (9.5)	
Family income	High	22 (19.6)	13 (22.8)	10 (13.5)	0.060
	Average	33 (29.5)	26 (45.6)	23 (31.1)	
	Low	57 (50.9)	18 (31.6)	41 (55.4)	

* Chi-square test.

**Table 2:** Frequency and percentage distribution of prevalent IOTN-DHC grades/subgrades (n = 243).

Grades/Subgrades	n (%)
**Grade 1 (No Need)**	**30 (12.3)**
**Grade II (Little Need)**	**82 (33.7)**
a-Increased overjet > 3.5 mm ≤ 6 mm with competent lips	11 (4.5)
b-Reverse overjet > 0 mm ≤ 1 mm	7 (2.9)
c-Anterior/posterior crossbite ≤ 1 mm discrepancy	19 (7.8)
d-Contact point displacements > 1 mm ≤ 2 mm	22 (9.1)
e-Anterior/posterior openbite > 1 mm ≤ 2 mm	16 (6.6)
f-Increased overbite ≥ 3.5 mm without gingival contact	5 (2.1)
**Grade III (Moderate Need)**	**57 (23.5)**
a-Increased overjet > 3.5 mm but ≤6 mm with incompetent lips	4 (1.6)
b-Reverse overjet > 1 mm but ≤3.5 mm	3 (1.2)
c-Anterior/posterior crossbites > 1 mm ≤ 2 mm	19 (7.8)
d-Contact point displacements > 2 mm ≤ 4 mm	9 (3.7)
e-Lateral/anterior open bites > 2 mm ≤ 4 mm	24 (9.9)
**Grade IV (Great Need)**	**52 (21.4)**
a-Increased overjet > 6 mm ≤ 9 mm	10 (4.1)
c-Anterior/posterior crossbites > 2 mm	14 (5.8)
d-Severe contact point displacements > 4 mm	12 (4.9)
e-Extreme lateral or anterior openbites > 4 mm	9 (3.7)
f-Increased/complete overbite with gingival/palatal trauma	3 (1.2)
h-Less extensive hypodontia requiring pre-restorative orthodontics or orthodontic space closure	4 (1.6)
**Grade V (Severe Need)**	**22 (9.1)**
a-Increased overjet > 9 mm	3 (1.2)
I-Impeded eruption of teeth.	18 (7.4)
s-Submerged deciduous teeth	1 (0.4)

**Table 3:** Comparison of mean OHIP-14 score across demographic variables.

		N	Mean ± SD	Mean Rank	K-WH/MU	df	*p*
Gender	Female	119	10.94 ± 8.17	133.13	6054.00	-	0.015 *
	Male	124	8.44 ± 7.15	111.32			
Educational level	Secondary	143	9.72 ± 7.67	122.95	0.648	2	0.723
	High school	77	9.82 ± 7.71	123.58			
	College	23	8.83 ± 8.67	110.83			
Family Income	High	45	8.22 ± 6.71	110.01	6.656	2	0.036 *
	Average	82	10.68 ± 8.30	130.99			
	Low	116	11.53 ± 8.67	136.51			

K-WH = Kruskal–Wallis test, df = degress of freedom, * *p* < 0.05 (Mann–Whitney U test).

**Table 4:** Mean score of the individual and overall OHIP-14 items of the participants.

OHIP-14 Domains	OHIP-14 Items	Mean ± SD
Functional Limitations	OHIP Q1	0.79 ± 1.21
	OHIP Q2	0.30 ± 0.74
Physical Pain	OHIP Q3	1.53 ± 1.18
	OHIP Q4	1.00 ± 1.18
Physical Disability	OHIP Q5	0.31 ± 0.74
	OHIP Q6	0.26 ± 0.68
Psycological Discomfort	OHIP Q7	1.23 ± 1.47
	OHIP Q8	1.01 ± 1.34
Psycological Disability	OHIP Q9	0.86 ± 1.19
	OHIP Q10	0.89 ± 1.26
Social Disability	OHIP Q11	0.49 ± 0.98
	OHIP Q12	0.25 ± 0.66
Handicap	OHIP Q13	0.38 ± 0.88
	OHIP Q14	0.36 ± 0.84
	Overall OHIP-14	9.67 ± 7.75

**Table 5:** Mean ± SD overall OHIP-14 score among different types of IOTN-DHC groups.

OHIP-14 Domains	OHIP-14 Items	Little or No Treatment Need	Borderline Need	Treatment Required	*p*
Functional Limitations	OHIP Q1	0.60 ± 1.03	0.86 ± 1.20	1.04 ± 1.41	0.159
	OHIP Q2	0.25 ± 0.56	0.44 ± 0.91	0.28 ± 0.82	0.270
Physical Pain	OHIP Q3	1.41 ± 1.14	1.75 ± 1.14	1.53 ± 1.25	0.120
	OHIP Q4	0.89 ± 1.11	1.23 ± 1.23	1.00 ± 1.24	0.240
Physical Disability	OHIP Q5	0.32 ± 0.71	0.21 ± 0.62	0.36 ± 0.85	0.437
	OHIP Q6	0.23 ± 0.60	0.18 ± 0.54	0.35 ± 0.87	0.499
Psycological Discomfort	OHIP Q7	0.93 ± 1.31	1.33 ± 1.50	1.59 ± 0.60	0.019 *
	OHIP Q8	0.81 ± 1.28	1.07 ± 1.36	1.26 ± 1.39	0.062
Psycological Disability	OHIP Q9	0.61 ± 0.97	0.98 ± 1.20	1.15 ± 1.39	0.023 *
	OHIP Q10	0.59 ± 1.08	1.00 ± 1.27	1.27 ± 0.40	0.002 *
Social Disability	OHIP Q11	0.46 ± 0.89	0.53 ± 1.14	0.51 ± 0.56	0.912
	OHIP Q12	0.21 ± 0.62	0.37 ± 0.84	0.22 ± 0.56	0.295
Handicap	OHIP Q13	0.32 ± 0.77	0.44 ± 0.91	0.43 ± 1.02	0.711
	OHIP Q14	0.28 ± 0.62	0.28 ± 0.70	0.54 ± 1.16	0.558
	Overall scores	7.92 ± 6.90	10.67 ± 7.14	11.54 ± 8.88	0.006 **

* p < 0.05 (Kruskal–Wallis test); ** *p* < 0.05 (Mann–Whitney U test).

**Table 6:** Frequency of impact (in %) of each item of the OHIP-14 on IOTN-DHC in relation to the gender of the study participants (N = 243).

OHIP-14	Little or No Treatment Need			Borderline Need			Treatment Required		
	Female	Male	*p*	Female	Male	*p*	Female	Male	*p*
OHIP Q1	34.5	33.3	0.892	37	40	0.819	40.5	43.2	0.814
OHIP Q2	20	19.3	0.926	22.2	30	0.506	16.2	13.5	0.744
OHIP Q3	69.1	71.9	0.742	81.5	73.3	0.464	75.7	59.5	0.136
OHIP Q4	49.1	47.4	0.855	81.5	33.3	<0.001 *	56.8	40.5	0.163
OHIP Q5	25.5	15.8	0.206	14.8	10	0.580	24.3	13.5	0.235
OHIP Q6	20	12	0.266	7.4	13.3	0.467	18.9	16.2	0.760
OHIP Q7	41.8	40.4	0.875	40.7	56.7	0.230	59.5	51.4	0.483
OHIP Q8	40	33.3	0.464	37	50	0.325	67.6	37.8	0.010 *
OHIP Q9	38.2	31.6	0.463	40.7	53.3	0.342	51.4	43.2	0.485
OHIP Q10	36.4	24.6	0.174	40.7	50	0.483	62.2	40.5	0.063
OHIP Q11	34.5	24.6	0.247	18.5	30	0.315	32.4	21.6	0.295
OHIP Q12	16.4	10.5	0.365	25.9	20	0.594	18.9	10.8	0.327
OHIP Q13	18.2	17.5	0.930	14.8	30	0.173	21.6	18.9	0.773
OHIP Q14	25.5	12.3	0.074	14.8	16.7	0.848	29.7	13.5	0.090

* Statistically significant between the groups (*p* < 0.05).

**Table 7:** Frequency of impact (in %) of each item of the OHIP-14 on IOTN-DHC in relation to the educational level of the study participants (N = 243).

OHIP-14	Little or No Treatment Need				Borderline Need				Treatment Required			
	Secondary School	High School	College	*p*	Secondary School	High School	College	*p*	Secondary School	High School	College	*p*
OHIP Q1	60.5	26.3	13.2	0.721	68.2	31.8	0	*p* < 0.001 *	58.1	32.3	9.7	0.809
OHIP Q2	54.5	31.8	13.6	0.849	66.7	26.7	6.7	0.986	45.5	45.5	9.1	0.793
OHIP Q3	59.5	30.4	10.1	0.951	68.2	31.8	0	*p* < 0.001 *	54	34	12	0.519
OHIP Q4	59.3	31.5	9.3	0.882	71.9	21.9	6.3	0.44	63.9	30.6	5.6	0.217
OHIP Q5	56.5	34.8	8.7	0.85	85.7	14.3	0	*p* < 0.001 *	64.3	21.4	14.3	0.4
OHIP Q6	44.4	44.4	11.1	0.338	83.3	16.7	0	*p* < 0.001 *	53.8	30.8	15.4	0.695
OHIP Q7	56.5	32.6	10.9	0.901	75	21.4	3.6	0.265	51.2	43.9	4.9	0.168
OHIP Q8	51.2	34.	14.6	0.39	72	24	4	0.553	51.3	43.6	5.1	0.235
OHIP Q9	51.3	38.5	10.3	0.387	77.8	18.5	3.7	0.151	51.4	42.9	5.7	0.405
OHIP Q10	58.8	29.4	11.8	0.968	69.2	26.9	3.8	0.655	50	44.7	5.3	0.207
OHIP Q11	63.6	27.3	9.1	0.803	64.3	35.7	0	*p* < 0.001 *	55	40	5	0.714
OHIP Q12	46.7	46.7	6.7	0.329	76.9	23.1	0	0.426	54.5	27.3	18.2	0.512
OHIP Q13	50	30	20	0.318	61.5	38.5	0	0.395	33.3	46.7	20	0.12
OHIP Q14	57.1	33.3	9.5	0.94	88.9	11.1	0	0.248	56.3	43.8	0	0.326

* Statistically significant between the groups (*p* < 0.05).

**Table 8:** Frequency of impact (in %) of each item of the OHIP-14 on IOTN-DHC in relation to the family income of the study participants (N = 243).

OHIP-14	Little or No Treatment Need				Borderline Need				Treatment Required			
	High	Average	Low	*p*	High	Average	Low	*p*	High	Average	Low	*p*
OHIP Q1	21.1	26.3	52.6	0.867	9.1	40.9	50	0.031 *	16.1	25.8	58.1	0.665
OHIP Q2	4.5	54.5	40.9	0.008 *	26.7	46.7	26.7	0.864	18.2	36.4	45.5	758
OHIP Q3	15.2	31.6	53.2	0.182	25	47.7	27.3	0.421	16	28	56	0.553
OHIP Q4	14.8	37	48.1	0.181	28.1	46.9	25	0.381	13.9	38.9	47.2	0.328
OHIP Q5	8.7	43.5	47.8	0.153	42.9	42.9	14.3	0.337	21.4	35.7	42.9	0.494
OHIP Q6	16.7	22.2	61.1	0.632	33.3	50	16.7	0.662	23.1	30.8	46.2	0.519
OHIP Q7	28.3	23.9	47.8	0.141	21.4	46.4	32.1	0.971	19.5	34.1	46.3	0.129
OHIP Q8	24.4	26.8	48.8	0.622	32	44	24	0.289	20.5	30.8	48.7	0.161
OHIP Q9	20.5	23.1	56.4	0.546	25.9	40.7	33.3	0.765	17.1	37.1	45.7	0.278
OHIP Q10	26.5	20.6	52.9	0.286	34.6	38.5	26.9	0.151	15.8	31.6	52.6	0.813
OHIP Q11	15.2	36.4	48.5	0.525	28.6	50	21.4	0.621	15	20.0	65	0.453
OHIP Q12	13.3	33.3	53.3	0.795	23.1	69.2	7.7	0.078	18.2	45.5	36.4	0.384
OHIP Q13	20	30	50	0.996	38.5	46.2	15.4	0.201	20	60	20	0.007 *
OHIP Q14	9.5	42.9	47.6	0.226	22.2	66.7	11.1	0.293	25	31.3	43.8	0.287

* Statistically significant between the groups (*p* < 0.05).

## Data Availability

The data presented in this study are available upon request from the corresponding author.

## References

[1] Jenny J., Cons N.C. (1996). Establishing malocclusion severity levels on the Dental Aesthetic Index (DAI) scale. Aus. Dent. J..

[2] Kharbanda O. (1999). What is the prevalence of malocclusion in India? Do we know Orthodontic Treatment Needs of Our country?. Ind. Orthod. Soc..

[3] Mtaya M., Brudvik P., Astrøm A.N. (2009). Prevalence of malocclusion and its relationship with socio-demographic factors, dental caries, and oral hygiene in 12- to 14-year-old Tanzanian schoolchildren. Eur. J. Orthod..

[4] Cunningham S.J., Hunt N.P. (2001). Quality of life and its importance in orthodontics. J. Orthod..

[5] Inglehart M.R., Bagramian R. (2002). Oral Health-Related Quality of Life.

[6] Alrashed M., Alqerban A. (2021). The relationship between malocclusion and oral health-related quality of life among adolescents: A systematic literature review and meta-analysis. Eur. J. Orthod..

[7] Feu D., Miguel J.A., Celeste R.K., Oliveira B.H. (2013). Effect of orthodontic treatment on oral health-related quality of life. Angle Orthod..

[8] Ahmed B., Gilthorpe M.S., Bedi R. (2001). Agreement between normative and perceived orthodontic need amongst deprived multiethnic school children in London. Clin. Orthod. Res..

[9] Hunt O., Hepper P., Johnston C., Stevenson M., Burden D. (2002). The Aesthetic Component of the Index of Orthodontic Treatment Need validated against lay opinion. Eur. J. Orthod..

[10] Zhang M., McGrath C., Hägg U. (2006). The impact of malocclusion and its treatment on quality of life: A literature review. Int. J. Paediatr. Dent..

[11] Bedi R., Gulati N., McGrath C. (2005). A study of satisfaction with dental services among adults in the United Kingdom. Br. Dent. J..

[12] McGrath C., Bedi R. (1999). The value and use of ‘quality of life’ measures in the primary dental care setting. Prim. Dent. J..

[13] Masood M., Masood Y., Saub R., Newton J.T. (2014). Need of minimal important difference for oral health-related quality of life measures. J. Public Health Dent..

[14] Agou S., Malhotra M., Tompson B., Prakash P., Locker D. (2008). Is the child oral health quality of life questionnaire sensitive to change in the context of orthodontic treatment? A brief communication. J. Public Health Dent..

[15] Al-Emran S., Wisth P.J., Böe O.E. (1990). Prevalence of malocclusion and need for orthodontic treatment in Saudi Arabia. Community Dent. Oral Epidemiol..

[16] El-Mangoury N.H., Mostafa Y.A. (1990). Epidemiologic panorama of dental occlusion. Angle Orthod..

[17] Thilander B., Myrberg N. (1973). The prevalence of malocclusion in Swedish schoolchildren. Eur. J. Oral Sci..

[18] Cenzato N., Nobili A., Maspero C. (2021). Prevalence of Dental Malocclusions in Different Geographical Areas: Scoping Review. Dent. J..

[19] Lombardo G., Vena F., Negri P., Pagano S., Barilotti C., Paglia L., Colombo S., Orso M., Cianetti S. (2020). Worldwide prevalence of malocclusion in the different stages of dentition: A systematic review and meta-analysis. Eur. J. Paediatr. Dent..

[20] Brook P.H., Shaw W.C. (1989). The development of an index of orthodontic treatment priority. Eur. J. Orthod..

[21] Chen M., Feng Z.C., Liu X., Li Z.M., Cai B., Wang D.W. (2015). Impact of malocclusion on oral health-related quality of life in young adults. Angle Orthod..

[22] Hassan A.H. (2006). Orthodontic treatment needs in the western region of Saudi Arabia: A research report. Head Face Med..

[23] Tolessa M., Singel A.T., Merga H. (2020). Epidemiology of orthodontic treatment need in southwestern Ethiopian children: A cross sectional study using the index of orthodontic treatment need. BMC Oral Health.

[24] Al-Azemi R., Artun J. (2010). Orthodontic treatment need in adolescent Kuwaitis: Prevalence, severity and manpower requirements. Med. Princ. Pract..

[25] Mugonzibwa E.A., Kuijpers-Jagtman A.M., Van ’t Hof M.A., Kikwilu E.N. (2004). Perceptions of dental attractiveness and orthodontic treatment need among Tanzanian children. Am. J. Orthod. Dentofacial. Orthop..

[26] Souames M., Bassigny F., Zenati N., Riordan P.J., Boy-Lefevre M.L. (2006). Orthodontic treatment need in French schoolchildren: An epidemiological study using the Index of Orthodontic Treatment Need. Eur. J. Orthod..

[27] Borzabadi-Farahani A., Borzabadi-Farahani A., Eslamipour F. (2009). Malocclusion and occlusal traits in an urban Iranian population. An epidemiological study of 11- to 14-year-old children. Eur. J. Orthod..

[28] Gudipaneni R.K., Aldahmeshi R.F., Patil S.R., Alam M.K. (2018). The prevalence of malocclusion and the need for orthodontic treatment among adolescents in the northern border region of Saudi Arabia: An epidemiological study. BMC Oral Health.

[29] Gunatissa C.N., Pathirage S.L., Ratnayaka N. (2016). The prevalence of malocclusion and orthodontic treatment need among 15-year-old school children in Galle district in Sri Lanka: An epidemiological study. APOS Trends Orthod..

[30] Perillo L., Masucci C., Ferro F., Apicella D., Baccetti T. (2010). Prevalence of orthodontic treatment need in southern Italian schoolchildren. Eur. J. Orthod..

[31] Masood Y., Masood M., Zainul N.N., Araby N.B., Hussain S.F., Newton T. (2013). Impact of malocclusion on oral health related quality of life in young people. Health Qual. Life Outcomes.

[32] Hakami Z., Chung H.S., Moafa S., Nasser H., Sowadi H., Saheb S., Bokhari A.M., Anderson N.K. (2020). Impact of fashion braces on oral health related quality of life: A web-based cross-sectional study. BMC Oral Health.

[33] Hassan A.H., Amin Hel S. (2010). Association of orthodontic treatment needs and oral health-related quality of life in young adults. Am. J. Orthod. Dentofacial. Orthop..

[34] Uzarevic Z., Bulj A. (2021). Oral Health-Related Quality of Life among Croatian University Students. Int. J. Environ. Res. Public Health.

[35] Altouki N.H., Albrahim M.A., Hassan A.H., Natto Z.S., Alhajrasi M.K. (2020). Oral Health-Related Quality of Life of Saudi Young Adults with Vertical Discrepancies in Occlusion. Patient Prefer. Adherence.

[36] Simões R.C., Goettems M.L., Schuch H.S., Torriani D.D., Demarco F.F. (2017). Impact of Malocclusion on Oral Health-Related Quality of Life of 8–12 Years Old Schoolchildren in Southern Brazil. Braz. Dent. J..

[37] World Health Organization (2013). Oral Health Surveys: Basic Methods.

[38] Gomes M.C., Pinto-Sarmento T.C., Costa E.M., Martins C.C., Granville-Garcia A.F., Paiva S.M. (2014). Impact of oral health conditions on the quality of life of preschool children and their families: A cross-sectional study. Health Qual. Life Outcomes.

[39] Masood M., Suominen A.L., Pietila T., Lahti S. (2017). Malocclusion traits and oral health-related quality of life in Finnish adults. Community Dent. Oral Epidemiol..

[40] Kang J.-M., Kang K.-H. (2014). Effect of malocclusion or orthodontic treatment on oral health-related quality of life in adults. Korean J. Orthod..

[41] Alhummayani F.M., Taibah S.M. (2018). Orthodontic treatment needs in Saudi young adults and manpower requirements. Saudi Med. J..

[42] Al-Jobair A.M., Baidas L.F., Al-Hamid A.A., Al-Qahtani S.G., Al-Najjar A.T., Al-Kawari H.M. (2016). Orthodontic treatment need among young Saudis attending public versus private dental practices in Riyadh. Clin. Cosmet. Investig. Dent..

[43] Hassan A.H., Hassan M.H., Linjawi A.I. (2014). Association of orthodontic treatment needs and oral health-related quality of life in Saudi children seeking orthodontic treatment. Patient Prefer. Adherence.

[44] Al-Zubair N.M. (2014). Orthodontic treatment need of Yemeni children assessed with dental aesthetic index. J. Orthod. Sci..

[45] Dalaie K., Behnaz M., Khodabakhshi Z., Hosseinpour S. (2018). Impact of malocclusion severity on oral health-related quality of life in an Iranian young adult population. Eur. J. Dent..

[46] Burden D.J., Pine C.M., Burnside G. (2001). Modified IOTN: An orthodontic treatment need index for use in oral health surveys. Community Dent. Oral Epidemiol..

[47] Lanteri V., Cavagnetto D., Abate A., Mainardi E., Gaffuri F., Ugolini A., Maspero C. (2020). Buccal Bone Changes around First Permanent Molars and Second Primary Molars after Maxillary Expansion with a Low Compliance Ni-Ti Leaf Spring Expander. Int. J. Environ. Res. Public Health.

[48] Bilgic F., Gelgor I.E., Celebi A.A. (2015). Malocclusion prevalence and orthodontic treatment need in central Anatolian adolescents compared to European and other nations’ adolescents. Dent. Press J. Orthod..

[49] Ciuffolo F., Manzoli L., D’Attilio M., Tecco S., Muratore F., Festa F., Romano F. (2005). Prevalence and distribution by gender of occlusal characteristics in a sample of Italian secondary school students: A cross-sectional study. Eur. J. Orthod..

[50] Thilander B., Pena L., Infante C., Parada S.S., de Mayorga C. (2001). Prevalence of malocclusion and orthodontic treatment need in children and adolescents in Bogota, Colombia. An epidemiological study related to different stages of dental development. Eur. J. Orthod..

[51] Gherunpong S., Tsakos G., Sheiham A. (2006). A sociodental approach to assessing dental needs of children: Concept and models. Int. J. Paediatr. Dent..

[52] Sischo L., Broder H.L. (2011). Oral health-related quality of life: What, why, how, and future implications. J. Dent. Res..

[53] Rodakowska E., Mierzyńska K., Bagińska J., Jamiołkowski J. (2014). Quality of life measured by OHIP-14 and GOHAI in elderly people from Bialystok, north-east Poland. BMC Oral Health.

[54] Ikebe K., Watkins C.A., Ettinger R.L., Sajima H., Nokubi T. (2004). Application of short-form oral health impact profile on elderly Japanese. Gerodontology.

[55] Gonzales-Sullcahuamán J.A., Ferreira F.M., de Menezes J.V., Paiva S.M., Fraiz F.C. (2013). Oral health-related quality of life among Brazilian dental students. Acta Odontol. Latinoam..

[56] Isiekwe G.I., Onigbogi O.O., Olatosi O.O., Sofola O.O. (2014). Oral health quality of life in a Nigerian university undergraduate population. J. West Afr. Coll. Surg..

[57] Klages U., Bruckner A., Zentner A. (2004). Dental aesthetics, self-awareness, and oral health-related quality of life in young adults. Eur. J. Orthod..

[58] Silvola A.S., Rusanen J., Tolvanen M., Pirttiniemi P., Lahti S. (2012). Occlusal characteristics and quality of life before and after treatment of severe malocclusion. Eur. J. Orthod..

[59] Yetkiner E., Vardar C., Ergin E., Yücel C., Ersin N. (2013). Orthodontic Treatment Need, Self-Esteem, and Oral Health-Related Quality of Life Assessment of Primary Schoolchildren: A Cross-Sectional Pilot Study. Turk. J. Orthod..

[60] Anthony S.N., Zimba K., Subramanian B. (2018). Impact of Malocclusions on the Oral Health-Related Quality of Life of Early Adolescents in Ndola, Zambia. Int. J. Dent..

[61] Scapini A., Feldens C.A., Ardenghi T.M., Kramer P.F. (2012). Malocclusion impacts adolescents’ oral health–related quality of life. Angle Orthodo..

[62] Thiruvenkadam G., Asokan S., John J., Geetha Priya P., Prathiba J. (2015). Oral health-related quality of life of children seeking orthodontic treatment based on child oral health impact profile: A cross-sectional study. Contemp. Clin. Dent..

[63] De Oliveira C.M., Sheiham A. (2004). Orthodontic treatment and its impact on oral health-related quality of life in Brazilian adolescents. J. Orthod..

[64] Kavaliauskienė A., Šidlauskas A., Zaborskis A. (2018). Relationship Between Orthodontic Treatment Need and Oral Health-Related Quality of Life among 11–18-Year-Old Adolescents in Lithuania. Int. J. Environ. Res. Public Health.

[65] Baskaradoss J.K., Geevarghese A., Alsaadi W., Alemam H., Alghaihab A., Almutairi A.S., Almthen A. (2022). The impact of malocclusion on the oral health related quality of life of 11–14-year-old children. BMC Pediatr..

[66] Jeelani W., Fida M., Shaikh A. (2016). The duration of pubertal growth peak among three skeletal classes. Dent. Press J. Ortho..

[67] Wide U., Hakeberg M. (2018). Oral health-related quality of life, measured using the five-item version of the Oral Health Impact Profile, in relation to socio-economic status: A population survey in Sweden. Eur. J. Oral Sci..

[68] Guimarães S.P.A., Jorge K.O., Fontes M.J.F., Ramos-Jorge M.L., Araújo C.T.P., Ferreira E.F., Melgaço C.A., Zarzar P.M. (2018). Impact of malocclusion on oral health-related quality of life among schoolchildren. Braz. Oral Res..

